# Evaluation of MVCT imaging dose levels during helical IGRT: comparison between ion chamber, TLD, and EBT3 films

**DOI:** 10.1120/jacmp.v17i1.5774

**Published:** 2016-01-08

**Authors:** Jean‐Pierre Mege, Sun Wenzhao, Attila Veres, Guillaume Auzac, Ibrahima Diallo, Dimitri Lefkopoulos

**Affiliations:** ^1^ Service de Physique Médicale Gustave Roussy Cancer Campus Villejuif France; ^2^ Department of Radiation Physics Sun Yat‐sen University Cancer Center Guangzhou China; ^3^ Equal‐Estro Laboratory Villejuif France; ^4^ CESP‐INSERM U1018, Gustave Roussy Cancer Campus Villejuif France

**Keywords:** doses, MVCT, tomotherapy, imaging modes

## Abstract

The purpose of this investigation was to evaluate the dose on megavoltage CT (MVCT) images required for tomotherapy. As imaging possibilities are often used before each treatment and usually used several times before the session, we tried to evaluate the dose delivered during the procedure. For each scanning mode (fine, normal, and coarse), we first established the relative variation of these doses according to different technical parameters (explored length, patient setup). These dose variations measured with the TomoPhant, also known as Cheese phantom, showed the expected variations (due to the variation of scattered radiation) of 15% according to the explored length and ±5% according to the phantom setup (due to the variation of the point of measurement in the bore). In order to estimate patient doses, an anthropomorphic phantom was used for thermoluminescent and film dosimetry. The degree of agreement between the two methods was very satisfactory (the differences correspond to 5 mGy per imaging session) for the three sites studied (head & neck, thorax, and abdomen). These measurements allowed us to estimate the delivered dose of between 1 cGy and 4 cGy according to the site and imaging mode. Finally, we attempted to investigate a way to calculate this delivered dose in our patients from the study conducted on a cylindrical phantom and by taking into account data from the initial kV‐CT scan. The results we obtained were close to our measurements, with discrepancies below 5 mGy per MVCT.

PACS numbers: 87.53.Bn, 87.55.km, 87.55.Qr

## I. INTRODUCTION

More and more images are being used during current radiotherapy procedures to improve treatment quality and accuracy. Image‐guided radiotherapy (IGRT) allows us to take into account not only patient positioning and target localization, but also structure avoidance and morphological variations.^1^, ^2^, ^3^, ^4^, ^5^, ^6^ Different possibilities have been described to take into account patient management.^7^, ^8^ In order to have a comprehensive approach to the total delivered dose, it is important to evaluate these imaging doses during treatment procedures especially when these techniques are used frequently.

Different approaches have been published on how to evaluate such doses using kilovoltage or megavoltage cone‐beam computed tomography (kV‐CBCT or MV‐CBCT).^5^, ^9^, ^10^, ^11^, ^12^, ^13^, ^14^, ^15^, ^16^, ^17^, ^18^, ^19^

For helical CT scanners, the dose is evaluated from the ${\text{CTDI}}_{100}$ measurement (the integral of a single‐slice dose profile using a 100 mm long ion chamber). In order to evaluate the average dose, the ${\text{CTDI}}_{W}$ then takes into account peripheral doses.

Furthermore, before any helical treatment, the imaging mode is performed with a pitch value that is higher than 1 which causes heterogeneous dose distribution. Chen et al.^(20)^ showed that, because of the pitch used during image acquisition, the absorbed dose in a homogenous phantom will not be uniform but will exhibit a sinusoidal shape curve varying from 0.6 cGy up to 1.1 cGy. Chen and colleagues calculated that the ratio of the maximal dose to the center dose can reach a value of 4 for a 40 cm diameter phantom.

The aim of this work was, first, to study dose variation according to various parameters (pitch values, explored length, and phantom setup), and secondly, to perform dose measurements with an anthropomorphic phantom in order to establish dose levels for different anatomical sites (head & neck, thorax, and abdomen). We therefore chose to use the multislice average dose (MSAD) to evaluate the delivered dose.^(21)^

## II. MATERIALS AND METHODS

### A. Imaging device

**Figure 1:**
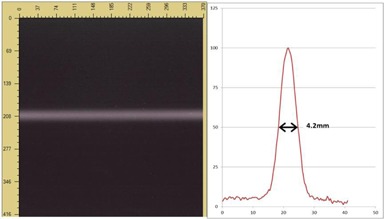
Imaging beam width evaluated with EBT3 film at the axis (performed in “calibration mode” to avoid any couch movement during irradiation).

Before a helical TomoTherapy (HT) (Accuray Inc, Madison, WI) procedure, an MVCT is performed in order to adjust the patient position. During imaging mode, the 6 MV linear accelerator is tuned to 3.5 MeV to improve image quality, and the collimator is set to obtain a 4 mm beam width. Measurements performed with EBT3 films in the TomoPhant, which is a cylindrical phantom (Fig. 1), show that, even if on the treatment console the MVCT scan mode sets the jaws to a position of ± 0.5 mm (called “J1”), the real imaging beam width is 4.2 mm at 85 cm from the source (at the isocenter).

Users only select the scan length from a sagittal plane and the reconstruction accuracy with a choice between three modes (fine, normal, and coarse). These three modes are obtained with different pitch values (the ratio of the couch travel per gantry rotation divided by the field width). In routine practice, these three modes are related to the couch speed (4 mm, 8 mm, and 12 mm per rotation). As the gantry period is set at 10 s in the imaging mode, the couch speed is then 24, 48, and 72 mm/min.

### B. Ionization chamber

Point‐dose measurements were performed with Exradin A1SL ion chamber (Standard Imaging, Middleton, WI) connected to the TomoElectrometer, which is the reference chain used during acceptance tests.

This chamber has a collecting volume of 0.053 cm^3^ (outside diameter od shell=6.35 mm, collector length=4.4 mm) and is calibrated in a ${\;}^{60}\text{Co}$ beam.

In the MVCT imaging mode, the energy of the incident electron beam is reduced in order to provide better imaging characteristics. Jeraj et al.^(22)^ reported an average energy of 1.5 MeV for the treatment mode and 1 MeV for the imaging mode.

Because of it, all MSAD are established without correction due to spectral changes in energy between the treatment and the imaging mode, as recommended by the TG‐148.^(23)^

AAPM TG‐51^(24)^ and TG‐148^(23)^ formalism were then used to evaluate the image doses:

(1) $$MSAD(Gcy)=H_{Q_{msr}}^{f_{msr}}\cdot N_{D,w,Q_{0}}\cdot k_{Q,Q_{0}}\cdot k_{Q_{msr}Q}^{f_{msr},f_{ref}}$$

where *Q* is the beam quality of the conventional reference field $f_{ref},Q_{msr}$ is the beam quality of the machine‐specific reference field $f_{msr},M_{Q_{msr}}^{f_{msr}}$ is the fully corrected electrometer reading, $N_{D,w,Q_{0}}$ is the ${\;}^{60}\text{Co}$ absorbed dose‐to‐water calibration factor determined by the ADCL (Accredited Dosimetry Calibration Laboratory, University of Wisconsin, Madison, WI), and $k_{Q,Q_{0}}\cdot k_{Q_{msr},Q}^{f_{msr}f_{ref}}$ is the quality conversion factor which accounts for the change in the absorbed dose‐to‐water calibration coefficient between the beam quality of interest and the ${\;}^{60}\text{Co}$ beam quality.

The coefficients we used to calculate the dose are determined according to TG‐148.^(23)^ We used a static 5 × 10 cm^2^ field (SSD 85 cm) to determine PDD (depth 10 cm) = 59.55%.

For $k_{Q,Q_{0}}\cdot k_{Q_{msr},Q}^{f_{msr}f_{ref}}$, we used a value of 0.998. As the image beam is a helical beam, according to TG‐148,^(23)^ a correction factor of 1.003 is applied.

Because of the duration of each irradiation (300 s, 150 s, and 100 s in the fine, normal, and coarse mode, respectively), the measured dose level and the setup (especially the beginning of the imaging zone), the reproducibility of our measurements is ± 0.2 cGy in the fine mode and ± 0.1 cGy in the normal and coarse mode. These values were evaluated with five different measurement of the central MSAD with our reference scanned length (10 cm).

### C. Gafchromic measurements

Gafchromic EBT3 films (Ashland Inc, Covington, KY) were evaluated using FilmQA Pro software (Ashland Inc.) with a flatbed scanner (Epson 11000XL, US Epson, Long Beach, CA) 24 hrs after the irradiation in order to stabilize their responses. To optimize our readings, and especially the flatbed scanner homogeneity, a template is centered on the surface to limit the zone of digitalization (which allows us a “landscape orientation” of ±20%). The film is placed under a 2 mm plate of glass placed above the films to hold them flat and the dose response is established according to the multichannel method.

As we use these films and software for pretreatment verifications, the response of our batch (A03031407) was established in a 6 MV standard beam (Clinac 21EX, Varian Medical Systems, Palo Alto, CA). The reference absorbed dose was determined according to the IAEA TRS 398 protocol^(25)^ with a NE 2571 chamber (0.6 cc) calibrated by our national laboratory ($\text{ND}=4.483\;10^{-2}\;\text{Gy}/\text{nC}$). The reference beam used for calibration (6 MV X‐ray beam), had a quality index defined as the ratio of dose at 200 mm and 100 mm from the surface, for a ±10% field in SAD conditions: ${\text{QI}=J}_{200}/J_{100}=0.675$.

The dose range is typically set from 0 to 2 Gy. With our routine film calibration, the mean accuracy was 3 cGy in the range of 10 to 50 cGy.

Different authors report low variations of response with energy for EBT films.^26^, ^27^, ^28^, ^29^, ^30^ Arjomandy et al.^(26)^ found less than ±9% of variation for EBT2 films on a photon energy range 75 kV to 18 MV. For EBT3, Villarreal‐Barajas and Khan^(30)^ and Casanova Borca et al.^(27)^ found less than 1% of variation between ${\;}^{60}\text{Co}$, 6 MV, and 18 MV photon beams on a dose range 50 to 400 cGy. Due to the low variation of response with energy,^26^, ^27^, ^28^, ^29^, ^30^ no specific correction was performed to take into account the variation between the reference beam used for calibration (6 MV X‐ray beam) and the beam used to perform imaging with the TomoTherapy unit.

To improve the accuracy of the evaluated dose, each film was irradiated several times to achieve a total dose of between 30 to 50 cGy (coarse mode: 25 times, normal mode: 15 times, fine mode: 10 times).

Because we have different conditions of irradiation and to facilitate the comparison between all detectors and modes, the dose distribution was recalculated by the software for only one acquisition and automatically analyzed.

### D. Thermoluminescent dosimeters

Lithium fluoride powder (LiF‐type Li‐7)‐based dosimeters (TLD700, Harshaw Chemical Company, Solon, OH) were used. The powder was encapsulated into opaque polyethylene cylindrical capsules measuring about 18 mm inner length, 3 mm inner diameter, and 1 mm thick walls. Each dosimeter contained about 160 mg of powder, allowing five readings per measurement point (about 30 mg per reading). The TLD signal reading was performed with a PCL‐3 (Fimel, Velizy, France) automated TLD reader. All TLDs were prepared and read by the Equal‐Estro Laboratory (Equal‐Estro, Villejuif, France).

**Figure 2:**
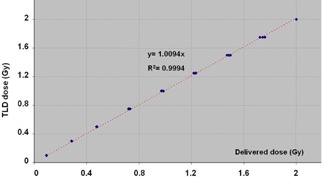
TLD700 dose response.

In order to determine the absorbed dose to water, a TLD calibration coefficient was applied to the TLDs signal. In imaging mode, the TLD calibration coefficient was the same as for the treatment mode. This coefficient was obtained during the external audit of our TomoTherapy unit by the Equal‐Estro laboratory (French regulations) for a dose of 2 Gy. No supplementary correction was applied to the TLD signal due to any potential change in response arising from the spectral change of the energy between the treatment and the imaging mode. The linearity of TLD dose response was checked in the range 0.1 to 2 Gy (Fig. 2).

### E. Phantoms

Two kinds of phantoms were used for this study. First, the TomoPhant (Gammex Inc., Middleton, WI), which is a cylindrical phantom with a 30 cm diameter and 18 cm in width with different holes for placing A1SL ion chambers along its diameter.

Ion chamber measurements were performed 5 mm from the center of this phantom (central doses) and 1 cm from the surface (peripheral dose). Films were used horizontally at the midplane

In the second part of our study, we used an anthropomorphic male ATOM phantom (CIRS Inc, Norfolk, VA), which is a cross‐sectional phantom in design with traditional 25 mm thick sections constituted by tissue‐equivalent epoxy resins which were used to simulate all anatomical parts of the body.

Anthropomorphic measurements were performed by film located vertically between two slices and by TLDs located at the machine isocenter, itself located at the center of each anatomical part.

### F. Dose prediction

Chen et al.^(20)^ published a simple formula to predict the imaging dose according to the mode and the diameter of a homogeneous cylindrical phantom. Cylindrical equivalence with an anthropomorphic phantom can be difficult due to the 3D shape of the contours and also the presence of heterogeneities (lung or bone). To overcome these difficulties, we used the AAPM report 220^(31)^ which shows different ways to calculate the equivalent patient size during CT examinations. Even if these approaches are established for low energies, we used them, in our particular conditions (MV images), to know if this methodology can help us to predict the dose levels taking into account heterogeneities and the shape of our patients.

We compared our anthropomorphic results to calculations obtained with our reference MSAD (from our cylindrical phantom) corrected by various approaches:

1. The smallest dimension between the height and width of a slice. In that case, heterogeneities will not be taken into account, nor the shape of the patient;
2. The effective diameter described as (AP+Lat)/2 or as $\sqrt{AP\times Lat}$ (AAPM report 220^(31)^). In that case, heterogeneities will not be taken into account, but the shape of the patient will be better modeled; or
3. The “water equivalent diameter” (WED), as defined in the AAPM report 220,^(31)^ taking into account heterogeneities and the patient shape:
  (2) $$WED(cm)=2\sqrt{\frac{A_{ROI}}{\pi }(1+\frac{\bar{CT(x,y)ROI}}{1000})}$$
  where $A_{ROI}$ is the area of the rectangular region of interest (ROI) that fully contains the slice, and $\bar{CT{\left(x,y\right)}_{ROI}}$ is the mean CT number in the ROI.

## III. RESULTS

### A. Imaging dose measurements

**Figure 3:**
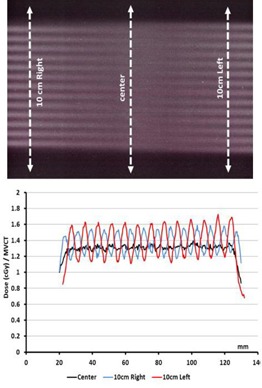
Results of 10 consecutive imaging irradiations (normal mode, 10 cm length). EBT3 films located in the middle of the TomoPhant and dose profiles at the center (black) and at 10 cm (red and blue).

All measurements are performed in clinical mode to evaluate the doses in a realistic situation and to avoid parameter variation (e.g., gantry rotation or couch movements) which is available in “calibration mode”. Figure 3 shows the irradiation obtained for a 10 cm length in a “normal mode” (couch speed=48 mm/min,pich value=2). This irradiation was performed 10 times with the same starting point in order to increase the dose measured by film. At the center of the irradiated zone, the measured dose corresponded to 1.3 cGy for 1 MVCT. Profiles were evaluated at the center and at 10 cm on both sides of the center of the phantom. The dose profile at the center was quite homogeneous along the 10 cm, but at 10 cm from the center we noticed that, first, the dose was 30% higher on the right or left side, second, the profiles showed sinusoidal shapes, and third, with an 8 mm period (corresponding to a 4 mm beam width with a pitch value of 2). The scanned length, evaluated in the central dose as the width of the 50% isodose was then 10.9 cm and the width of the 95% isodose was 10 cm.

We evaluated different point doses to obtain a weighted MSAD index (${\text{MSAD}}_{W}$) using the Exradin A1SL ion chamber and the TomoPhant. The machine isocenter was set at the center of the phantom.

(3) $$MSAD_{W}=\frac{D_{center}}{3}+\left(\frac{2}{3}\times D_{periphery}\right)$$

where $D_{center}$ is the dose in the center of the TomoPhant, and $D_{periphery}$ is the mean value calculated at 1 cm from the phantom surface for four measurement points (Table 1) in an acquired image measuring 10 cm in length.

If we compare the doses obtained with the A1SL ion chamber and those obtained with EBT3 films, we can see that both the central doses and peripheral doses are very close in normal mode (1.4 cGy vs. 1.3 cGy for the central dose and 1.7 cGy vs. 1.8 cGy for peripheral doses). Due to its sensitive length, the A1SL ion chamber integrates the dose throughout image acquisition and thus produces a lower dose. If we use film dosimetry and study the mean dose in a region of interest of 4 mm in length, the peripheral dose is then 1.5 cGy. This shows that the ion chamber can be used in the central zone where the homogeneity is better for evaluating the dose during MVCT.

The values obtained for the doses, the ratio of the average peripheral to the central dose, and ${\text{MSAD}}_{W}$ (Table 1) were very similar to published values^11^, ^32^ considering phantom and jaws settings variations and measurement conditions (Table 2).

**Table 1:** Doses and ${\text{MSAD}}_{W}$ (cGy) measured in the TomoPhant.

	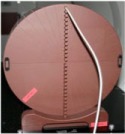	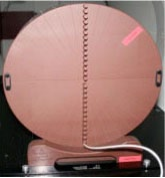	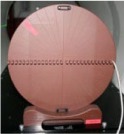	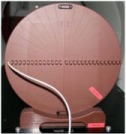	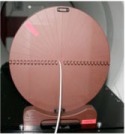			
*Mode*	*Anterior*	*Posterior*	*Left*	*Right*	*Central Dose*	*Maximal Dose/Central Dose*	*Average Peripheral/Central Dose*	${\text{MSAD}}_{W}$ *(cGy)*
Fine Pitch=1	3.4±0.2	3.1±0.2	3.1±0.2	3.3±0.2	2.7±0.2	1.26	1.19	3.0±0.2
Normal Pitch=2			1.7±0.1	1.7±0.1	1.4±0.1			
	1.5±0.1	1.6±0.1	1.8	1.8	1.3	1.21	1.16	1.5±0.1
			(EBT3)	(EBT3)	(EBT3)			
Coarse Pitch=3	0.8±0.1	1.5±0.1	1.0±0.1	1.2±0.1	1.0±0.1	1.5	1.13	1.1±0

**Table 2:** Comparison of different published values of the central dose according to detector type and irradiation conditions.

*Mode*	*Jung* ^(32)^	*Shah* ^(33)^		*Fast* ^(11)^	*Chen* ^(20)^	*Our Measurements*
Detector	A1SL 0.053 cm^3^ Standard Imaging	A1SL 0.053 cm^3^ Standard Imaging		M31002 0.125 cm^3^ PTW	Calculations	A1SL 0.053 cm^3^ Standard Imaging
Phantom	Virtual water Diameter 30 cm	Acrylic Diameter 20 cm	Virtual water Diameter 30 cm	Acrylic Diameter 33 cm	Acrylic and water Various diameters	Virtual water Diameter 30 cm
Scanned length	10.8 cm		18 cm		9.6 cm	10 cm
Fine (Pitch=1)	2.69 cGy	2.21 cGy		2.4 cGy	2.33 cGy	2.7 cGy
Normal (Pitch=2)	1.3 cGy	1.14 cGy	1.06 cGy	1.2 cGy	1.17 cGy	1.4 cGy
Coarse (Pitch=3)	0.85 cGy	0.76 cGy		0.8 cGy	0.78 cGy	1 cGy

Compared with the calculated values (Chen et al.^(20)^), we found higher values for the central doses (around +0.3 cGy in our conditions) and for the ratio of the peripheral dose to center dose for ion chamber measurements. (Our mean value of this ratio was 1.16 and Chen and colleagues calculated a value of 1.13). Concerning the ratio between the maximal dose to center dose, as we only evaluated it with four peripheral points located at the same slice position, whereas the Chen study took into account the entire volume examined, we had the greatest discrepancies. (Our ratios were 1.26, 1.21, and 1.5 and, as the variations are not linear, we estimate the ratios from the Chen study to be around 1.25, 1.6, and 1.8 for the fine, normal, and coarse mode, respectively).

By analogy with CT scanners for which the ${\text{CTDI}}_{\text{vol}}$ are obtained by dividing the CTDI value by the pitch value, we noted that, as expected, the MSAD in the fine mode was threefold higher than in the coarse mode and twofold higher than in the normal mode (pitch values 1, 2, and 3).

### B. Relative dose variation with the scanned length

In order to evaluate the dose variation in the clinical situation, different scanned lengths were studied. The center of the phantom was always set at the isocenter of the TomoTherapy unit.

**Figure 4:**
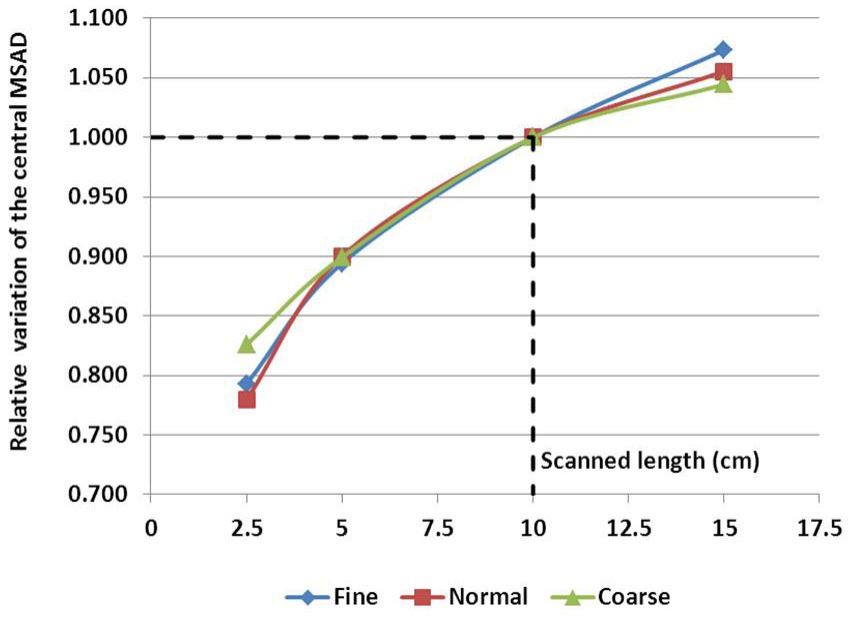
Central point MSAD variation according to the scanned length. The dose evaluation plan is always in the middle of the scanned length.

For each imaging mode (coarse, normal, and fine), we established the relative variation of the central MSAD according to the scanned length (Fig. 4).

As expected, due to the increase in scattered radiation, the central dose increased, but we found that the relative variation at the center of the phantom did not give rise to a significant discrepancy according to the imaging mode (fine, normal, or coarse). Unfortunately, because of the limited width of the TomoPhant (18 cm), no higher lengths were evaluated.

### C. Relative variation of the central MSAD with initial setup

**Figure 5:**
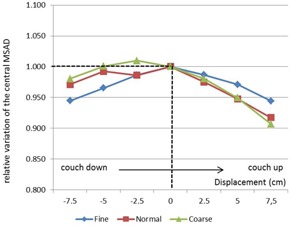
Relative variation of central dose with a different vertical offset.

With a TomoTherapy unit, only one direction (the height of the couch in the bore) can vary significantly from one patient to another. In order to quantify the influence of this setup on the central dose point (Fig. 5), we measured the dose in the three scanning modes with different distances between the beam isocenter and the center of the TomoPhant. The measurement point was always set at the center of the cylindrical phantom.

In these conditions, the dose variation also seemed to be independent of the mode (coarse, normal or fine), but we observed a low variation with the couch height (‐5% to −10%). Because of the cylindrical shape of the TomoPhant, the maximum response was obtained when the ion chamber was located at the isocenter.

## IV. DISCUSSION

### A. Measurements in an anthropomorphic phantom

In order to evaluate the dose in “clinical cases”, the ATOM‐CIRS phantom was used with radiochromic EBT3 films and thermoluminescent dosimeters to obtain more realistic conditions (patient size and shape) instead of a 30 cm diameter cylindrical phantom. We studied three different sites: head and neck (film located between slice # 8 and 9), thorax (film located between slice # 18 and 19), and abdomen (film located between slice # 23 and 24).

#### A.1 Films measurements

For each film, the phantom was set up on the couch like a patient with the machine isocenter located at mid‐thickness.

**Figure 6:**
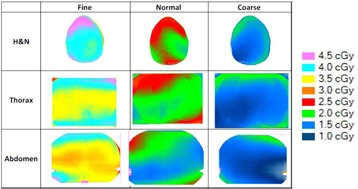
Measured dose distributions for 1 MVCT radiochromic EBT3+Film QA Pro for the three acquisition modes and three different sites (head & neck, thorax and abdomen).

Figure 6 shows the recalculated dose distributions for one irradiation. As expected, according to the selected scanning mode, different dose levels were obtained for each site and these dose levels are also related to patient thickness (higher doses for H&N with the fine mode and lower doses for abdomen with the coarse mode). Table 3 summarizes the results for the average MSAD and the MSAD range for the three sites and the three imaging modes obtained with film measurements. Between our values and those published by Shah et al.,^(33)^ we have large differences (+45% to +75%). This can be explained by the dose level, the type of results (films vs. calculations), the type of phantom (ATOM‐CIRS vs. real patients), beam calibration or software version.

Due to thickness variation, the doses evaluated with the anthropomorphic phantom were always higher than those obtained in the 30 cm diameter phantom because of an increase in the dose rate when the thickness decreased. Two effects must be taken into account regarding the ratio of the maximal dose to the central dose. First, this ratio will increase because of the effect of the percentage depth dose and, secondly, the dose will not be distributed homogeneously because of the pitch value (or the ripple dose distribution described by Chen et al.^(20)^). As expected, the dose increased when the acquisition mode was more precise and when the phantom thickness decreased.

The observed dose variations between our measurements in the anthropomorphic phantom and the cylindrical phantom show that the use of doses established in a standardized mode without correction factors can result in underestimation (30% for the head and neck and 10% for the abdomen).

**Table 3:** MSAD (for 1 MVCT) evaluated with radiochromic films for three anatomical regions (ATOM phantom) and comparison with the doses obtained in the cylindrical phantom. For comparison, results obtained by Shah et al.^(33)^ for different localizations.

	*Our Measurements*			*Shah^(333) Normal Mode^*
	*Fine*	*Normal*	*Coarse*
	*Head & Neck*			
Average MSAD (cGy)	3.9±0.3	2.1±0.2	1.4±0.1	1.45
MSAD range	[3.5‐4.5]	[1.5‐2.7]	[1‐1.8]	(parotids)
Maximal dose / Central dose	1.15	1.3	1.3	1.2
	*Thorax*			
Average MSAD (cGy)	3.5±0.3	2±0.2	1.3±0.1	1.14
MSAD range	[3 ‐ 4.1]	[1.5‐2.6]	[0.9‐2.1]	(lungs)
Maximal dose / Central dose	1.17	1.3	1.6	1.44
	*Abdomen*			
Average MSAD (cGy)	3.3±0.3	1.7±0.2	1.2±0.1	1.05
MSAD range	[2.8‐4.1]	[1.3‐2.3]	[0.7‐1.9]	(bladder)
Maximal dose / Central dose	1.24	1.35	1.6	1.25
Cylindrical phantom ϕ=30 cm	3	1.5	1.1	1.06
${\text{MSAD}}_{w}$ (cGy)				
Maximal dose / Central dose	1.26	1.21	1.5	1.08

To investigate the ripple effect, we tried to evaluate the variation in dose distribution along the scanned length. Three films were used simultaneously and located at 2.5 cm (interstices 17–18, 18–19, and 19–20) from the center (Table 4).

In fact, due to the thickness of each slice of the phantom (25 mm) and the couch speed (8 mm per rotation), the gantry rotated three times between each film, the geometrical conditions of the irradiation were very similar for the three films, resulting in comparable doses and dose distributions.

**Table 4:** MSAD variations for a 10 cm length MVCT (normal mode).

	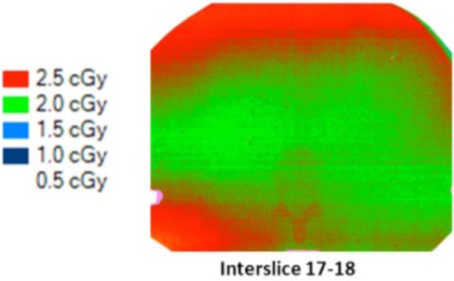	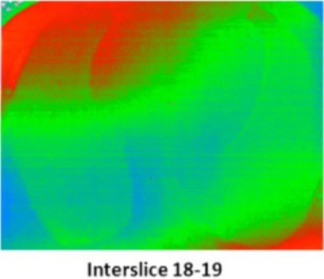	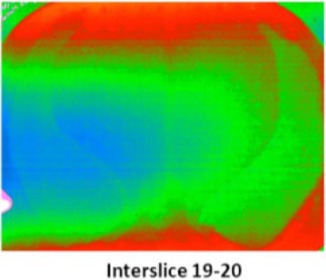
Thorax	Interslice 17‐18	Interslice 18‐19	Interslice 19‐20
*Thorax*	Interslice 17–18	Interslice 18–19	Interslice 19–20
Relative position	−2.5 cm	0	+2.5 cm
Central MSAD (cGy)	1.8	1.85	1.8
Average MSAD (cGy)	1.8	2	2
MSAD range	[1.3–2.3]	[1.5–2.7]	[1.4–2.8]

#### A.2 TLD measurements

TLD measurements were performed in the ATOM phantom placing the capsules in the dedicated holes (5 mm diameter). Due to the capsule size, the dose is integrated along the total length of the capsule (18 mm). Ideally, the TLD signal/noise should be higher than 4, so two consecutive irradiations were performed to increase the dose level. In order to facilitate the comparison between the different dose measurement methods (radiochromic films and TLD), the results correspond to the delivery of a single imaging procedure.

**Figure 7:**
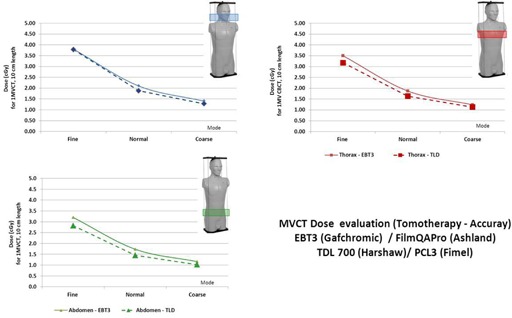
Comparison of measured doses in the ATOM phantom with TLD and radiochromic films for three different sites (evaluated point = center of the slice).

Because of the TLD length (18 mm), we choose to only estimate the dose in the central region of the slice where the delivered dose is the most homogeneous, detectors were then at the isocenter location. We found good agreement between TLD doses and radiochromic film doses (Fig. 7) for each acquisition mode, taking into account the dose levels, irradiation conditions (dose accumulation: twice for TLD and 10, 15, and 25 times for films for fine, normal, and coarse mode, respectively), the detector size, and phantom heterogeneities. Nevertheless, the TLD dose measurements were always lower, probably due to their elongated shape (18 mm length) which heterogeneously integrates a delivered dose. The agreement between TLD and Gafchromic films was between 0.2 cGy (head & neck) and 0.4 cGy (abdomen).

**Figure 8:**
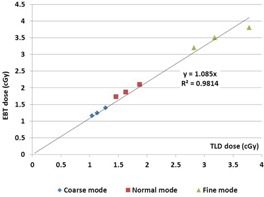
Comparison of measured doses between EBT3 and TLD according to the selected imaging mode.

Because of the hypotheses for dose determination without correction factors due to energy variation and differences in dose acquisition (2 examinations for TLD and between 10 and 25 examinations for films), the comparison between the doses obtained by film dosimetry and TLD (Fig. 8) shows that the difference is constant and of the order of 8.5%, with TLD always exhibiting the lowest doses.

#### B. Dose prediction

Using the Chen study's dose calculation formula,^(20)^ the three equivalent patient size estimations (AAPM report 220^(31)^) and only taking into account the smallest dimension between the height and width of a slice, we tried to find the equivalent patient size estimation that was the most appropriate for evaluating the dose during MVCT imaging. Table 5 shows the main characteristics of each slice we used for our study.

The agreement between the different approaches and our experimental data (Table 6) was always very good (less than 0.5 cGy, except for the thorax, probably because of the lung heterogeneities).

As mentioned in the AAPM report 220,^(31)^ the WED calculation requires the mean CT number which can be evaluated using tools available on planning stations, but this signifies manual and not practicable intervention. This is why we tried to find a simple and easy method to estimate the imaging dose during IGRT based on the smallest size of each slice.

**Table 5:** Main characteristics of each slice used (ATOM phantom).

	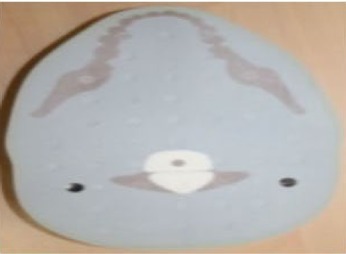	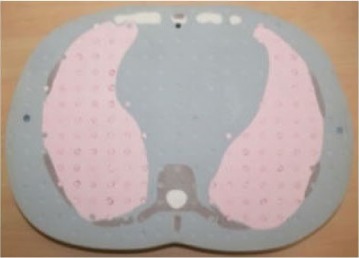	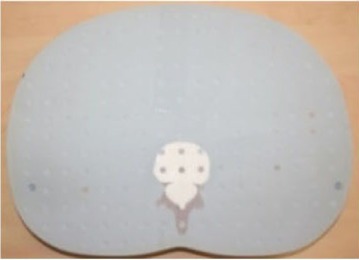
	*H&N Slices 8–9*	*Thorax Slices 18–19*	*Abdomen Slices 23–24*
Size: AP (cm) / Lat (cm)	17.8 / 12.3	23.5 / 31.9	22.3 / 28.5
Heterogeneity	Bone (vertebra and jaw)	Lung and Bone (vertebra)	Bone (vertebra)
$A_{\text{ROI}}({\text{cm}}^{2})$	219	750	636
$\bar{CT{\left(x,y\right)}_{ROI}}$	‐212	‐371	‐140
AAPM report 220: (AP+Lat)/2(cm)	15	27.7	25.5
$\sqrt{AP\times Lat}\;(\text{cm})$	14.8	27.4	25
WED (cm)	14.8	24.5	26.4
Smallest dimension (cm)	12.3	23.5	22.3

**Table 6:** MSAD calculated according to various hypotheses for the equivalent diameter using the formula by Chen et al.^(20)^ (in brackets, values corrected according to our A1SL measurements).

		*MSAD (cGy) Calculated From Chen* ^(20)^, ^a^ *Equivalent Patient Size*				*Our Measurements (cGy)*	
		(AP+Lat)/2	$\sqrt{AP\times Lat}$	*Water‐Equivalent Diameter*	*Smallest Dimension*	*Films*	*TLD*
H&N	Fine	3.5 (3.9)	3.55 (4)	3.55 (4)	3.7 (4.2)	3.9	3.9
Slices	Normal	1.75 (2.05)	1.8 (2.1)	1.8 (2.1)	1.85 (2.15)	2.1	1.8
8–9	Coarse	1.2 (1.5)	1.2 (1.5)	1.2 (1.5)	1.2 (1.5)	1.4	1.3
Thorax	Fine	2.7 (3)	2.7 (3)	2.9 (3.3)	2.95 (3.3)	3.5	3.1
Slices	Normal	1.35 (1.6)	1.35 (1.6)	1.45 (1.7)	1.5 (1.75)	2	1.6
18–19	Coarse	0.9 (1.1)	0.9 (1.1)	1 (1.2)	1 (1.2)	1.3	1.15
Abdomen	Fine	2.85 (3.2)	2.9 (3.25)	2.8 (3.15)	3.05 (3.4)	3.3	3.2
Slices	Normal	1.4 (1.6)	1.45 (1.7)	1.4 (1.6)	1.5 (1.75)	1.7	1.4
23–24	Coarse	0.95 (1.2)	0.95 (1.2)	0.95 (1.2)	1 (1.25)	1.2	1
			φ = 30 cm		30.3 cm	A1SL Ion Chamber	
Tomo	Fine		2.4		2.5	2.7±0.2	
“Cheese”	Normal		1,2		1.25	1.4±0.1	
Phantom	Coarse		0.8		0.8	1±0.1	

^a^ For water phantoms, calculations were performed as follows: Fine mode→dose(cGy)=4.52‐(0.066×diameter); Normal mode→dose(cGy)=2.26‐(0.033×diameter); Coarse mode→dose(cGy)=1.51‐(0.022×diameter).

In each experimental condition, our results were close to the expected values when we selected the smallest dimension of the phantom. Whatever the method used for the equivalent patient size, the calculated MSAD was very close to the others. In order to take into account our machine data, we recalculated (Table 6, values in brackets) the different MSAD with the ratio of the MSAD we obtained in the cylindrical phantom (ion chamber) to the MSAD given in the Chen study for each imaging mode. In that case, the MSAD, calculated either with the WED or the smallest dimension, were closer to our measurements (around 0.2 cGy).

This approach, based on a simple dose acquisition (cylindrical phantom, central MSAD), can be easily used to predict realistic imaging patient doses during helical IGRT.

### V. CONCLUSIONS

This work pinpoints various sources of dose variations during image acquisition in helical radiotherapy. The dose levels should be taken into account when establishing protocols, but we can base ourselves on our measurements made on cylindrical phantoms and use various solutions to estimate, in a realistic way, absorbed doses in the explored zone (shortest dimension or water‐equivalent diameter).

From our reference conditions (a helix measuring 10 cm in length centered on the gantry axis), the dose varies from ±10% according to the explored length and should be taken into account. Variations according to the patient setup (±5%) are less important and more difficult to estimate.

Our results, either in a cylindrical phantom with an ionization chamber, or in an anthropomorphic phantom with thermoluminescent detectors or EBT3 films, are in good agreement with those published in the literature whatever the imaging mode (fine, normal, coarse). Furthermore, they are also in good agreement with the results obtained by calculation on cylindrical phantoms.

The dose and its homogeneity are very dependent on the pitch and the site, but are around 4 cGy for the highest delivered dose and around 1 cGy for the lowest. Due to the imaging technology used in TomoTherapy procedures, the dose is the highest for head and neck (smallest thicknesses) treatments and decreases for thorax or abdomen.

Whatever the imaging mode chosen, frequent image acquisition can increase the dose significantly. In spite of the large number of sources of uncertainties (imaging beam difficult to characterize, low doses measurements, size of the detector, reproducibility of images acquisition), we can deduct, from few data measured in simple conditions (cylindrical phantom), levels of dose delivered to our patients during the imaging session. These doses are calculable by using published study (Chen et al.^(20)^) on one hand and the AAPM report 220^(31)^ on the other hand. They can be easily reported in our Record and Verify system (MOSAIQ; ELEKTA AB, Stockholm, Sweden) in order to document these procedures.

Furthermore, the knowledge of these dose levels, allows a justification of the imaging process and an adaptation of the conditions of acquisition to the therapeutic purpose. The use of the most irradiant mode can justify itself for certain cases as irradiations of small anatomical regions or the use of MVCT images for adaptive radiotherapy to improve the precision of repositioning or contouring.

It would be useful to compare the information provided by the images according to the various acquisition modes and to be able to choose, in a objective way, the most adapted IGRT protocol (frequency and mode) in order to limit the received doses while improving the quality of treatments.^5^, ^8^, ^15^, ^17^

Flynn^(34)^ pointed out that a small dose can change the treatment response and it seems important not to exceed 5% of the prescribed dose when the image is acquired few minutes before the irradiation. This dose (i.e., two or three sets of images) can be easily reached during IGRT when managing anatomical changes (e.g., rectal/bladder filling).

## Supporting information

Supplementary Material

Supplementary Material

Supplementary Material

Supplementary Material

Supplementary Material

Supplementary Material

Supplementary Material

Supplementary Material

Supplementary Material

Supplementary Material

Supplementary Material

Supplementary Material
