# Supplementary figures and images for: Evaluation of MVCT imaging dose levels during helical IGRT: comparison between ion chamber, TLD, and EBT3 films
Source: J Appl Clin Med Phys. 2016 Jan 8;17(1):143–57. doi: 10.1120/jacmp.v17i1.5774 (PMC5690206; doi:10.1120/jacmp.v17i1.5774)

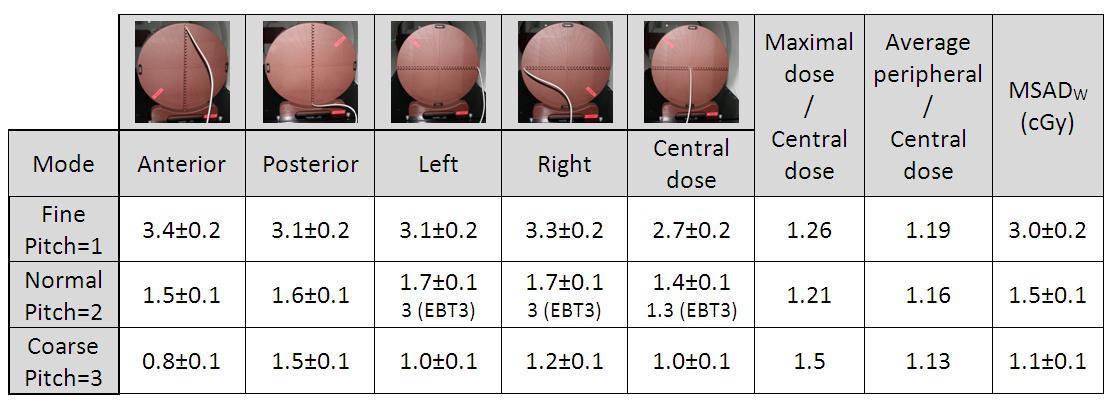

Supplement: Supplementary file 1 — Supplementary Material [file ACM2-17-143-s001.jpg]

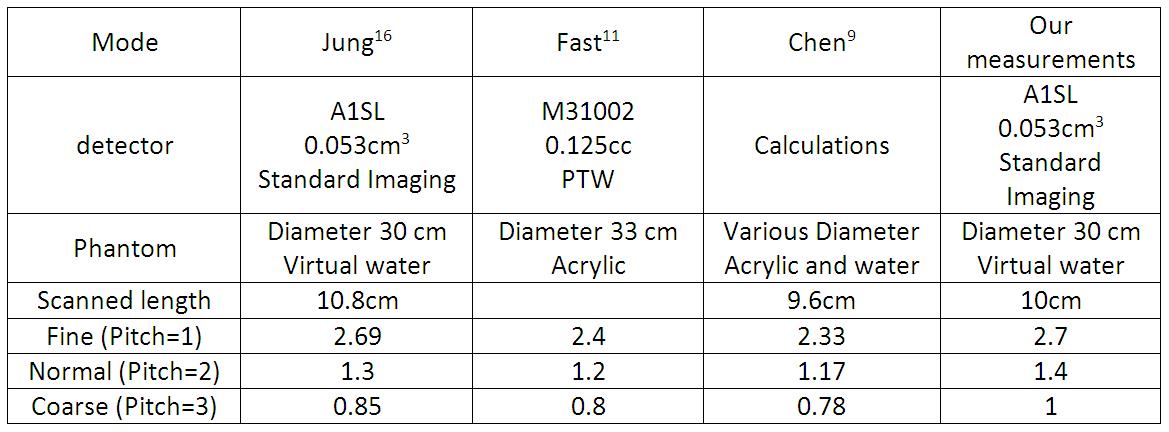

Supplement: Supplementary file 2 — Supplementary Material [file ACM2-17-143-s002.jpg]

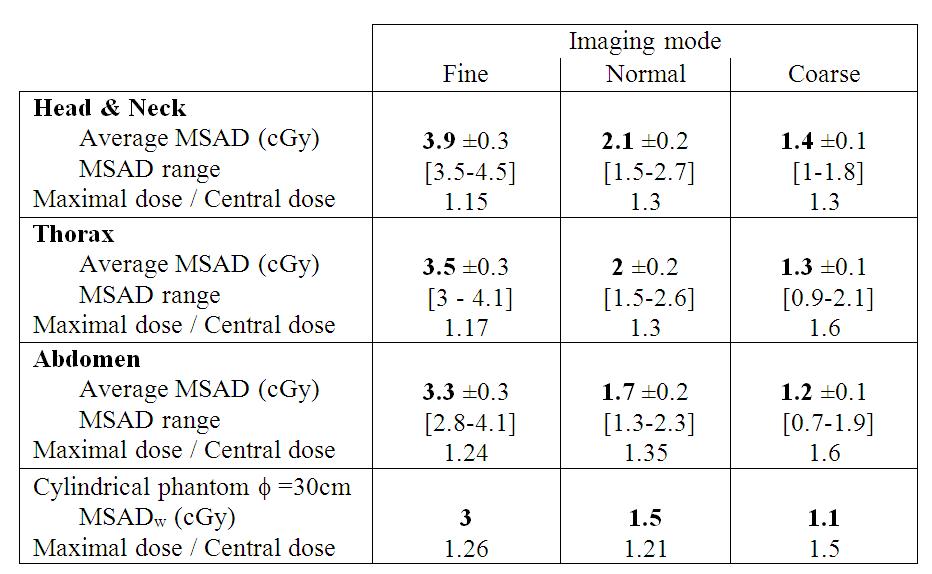

Supplement: Supplementary file 3 — Supplementary Material [file ACM2-17-143-s003.jpg]

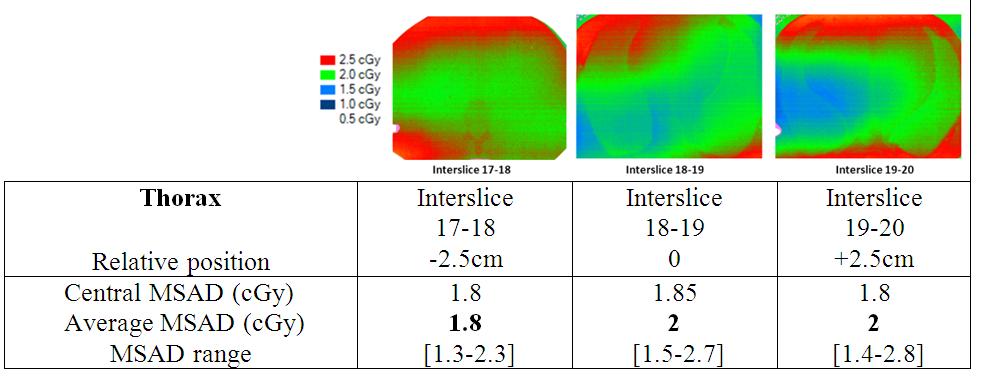

Supplement: Supplementary file 4 — Supplementary Material [file ACM2-17-143-s004.jpg]

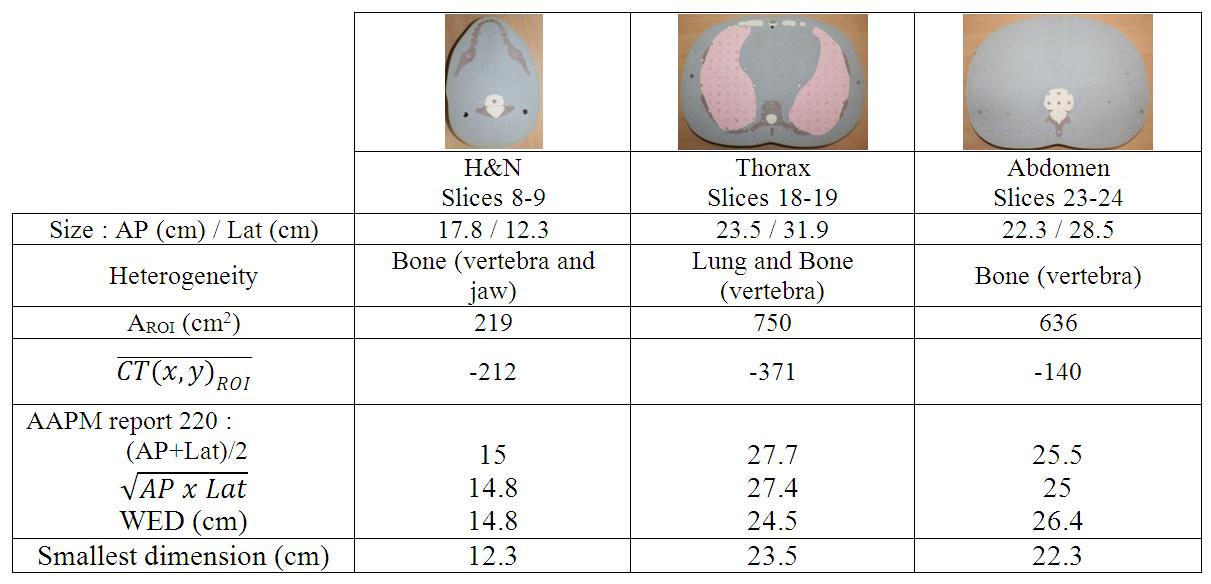

Supplement: Supplementary file 5 — Supplementary Material [file ACM2-17-143-s005.jpg]

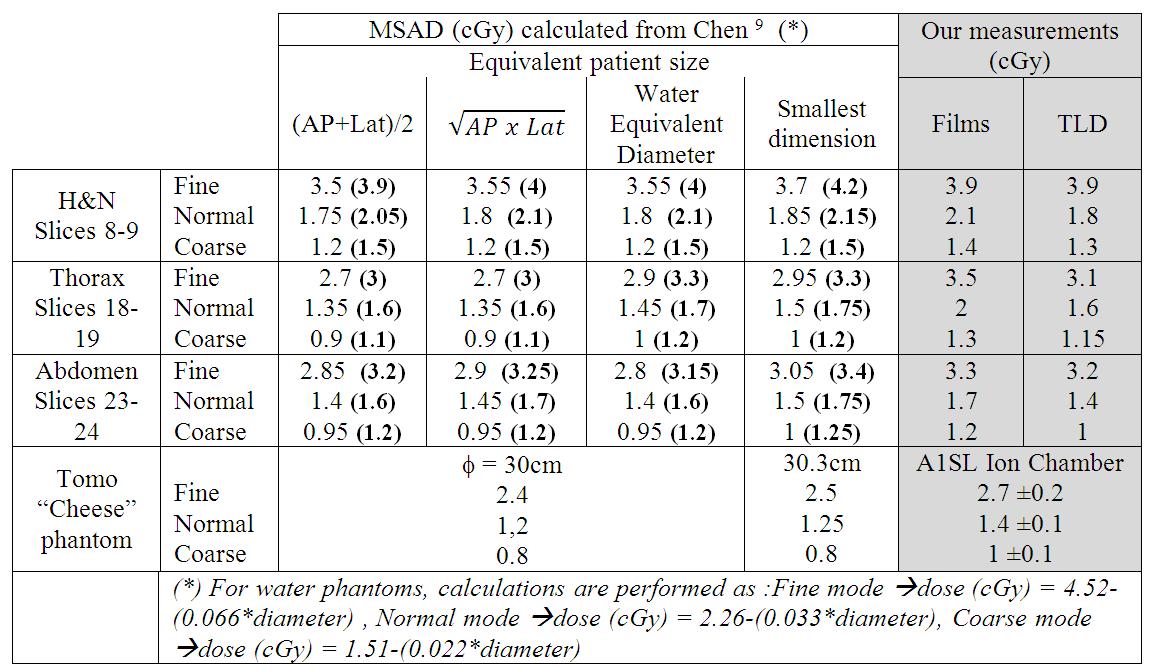

Supplement: Supplementary file 6 — Supplementary Material [file ACM2-17-143-s006.jpg]

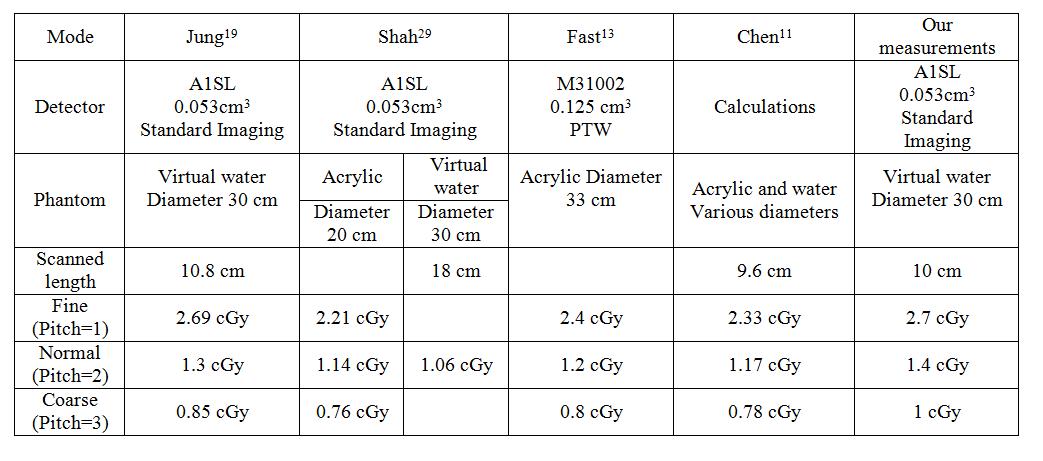

Supplement: Supplementary file 8 — Supplementary Material [file ACM2-17-143-s008.jpg]

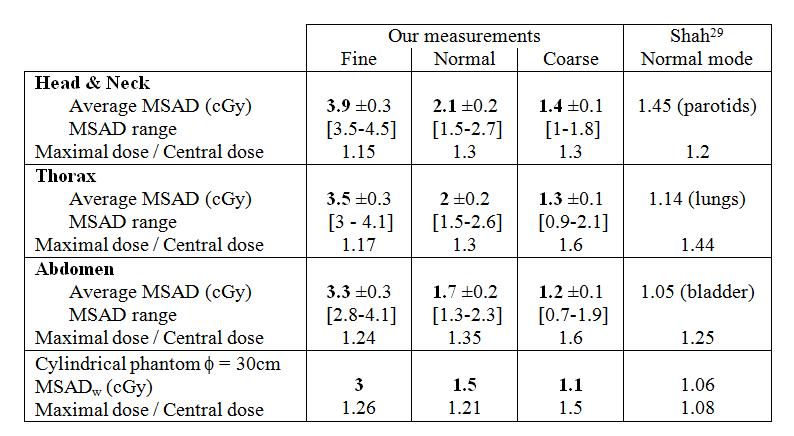

Supplement: Supplementary file 9 — Supplementary Material [file ACM2-17-143-s009.jpg]

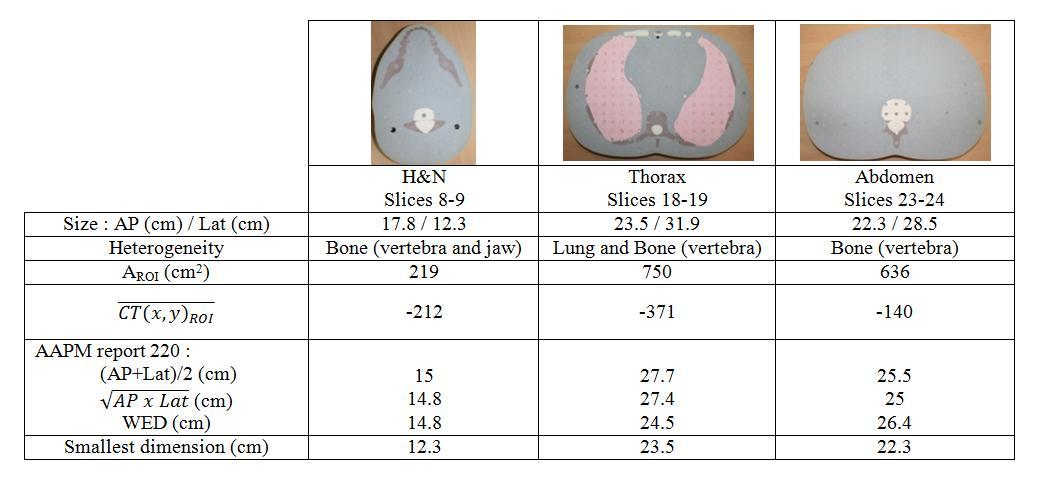

Supplement: Supplementary file 11 — Supplementary Material [file ACM2-17-143-s011.jpg]
